# Biomonitoring of 19 Mycotoxins in Plasma from Food-Producing Animals (Cattle, Poultry, Pigs, and Sheep)

**DOI:** 10.3390/toxins15040295

**Published:** 2023-04-18

**Authors:** Borja Muñoz-Solano, Elena González-Peñas

**Affiliations:** Department of Pharmaceutical Technology and Chemistry, Faculty of Pharmacy and Nutrition, Universidad de Navarra, 31008 Pamplona, Spain; bmunoz.1@alumni.unav.es

**Keywords:** biomonitoring, animal plasma, mycotoxins, sterigmatocystin, LC-MS/MS

## Abstract

Mycotoxins are of great concern in relation to food safety. When animals are exposed to them, health problems, economic losses in farms and related industries, and the carryover of these compounds to animal-derived foods can occur. Therefore, control of animal exposure is of great importance. This control may be carried out by analyzing raw material and/or feed or through the analysis of biomarkers of exposure in biological matrixes. This second approach has been chosen in the present study. Firstly, a methodology capable of analyzing mycotoxins and some derivatives (AFB1, OTA, ZEA, DON, 3- and 15-ADON, DOM-1, T-2, HT-2, AFM1, STER, NEO, DAS, FUS-X, AFB2, AFG1, AFG2, OTB, and NIV) by LC-MS/MS in human plasma, has been revalidated to be applied in animal plasma. Secondly, this methodology was used in 80 plasma samples obtained from animals dedicated to food production: cattle, pigs, poultry, and sheep (20 samples of each), with and without being treated with a mixture of β-glucuronidase-arylsulfatase to determine possible glucuronide and sulfate conjugates. Without enzymatic treatment, no mycotoxin was detected in any of the samples. Only one sample from poultry presented levels of DON and 3- and 15-ADON. With enzymatic treatment, only DON (1 sample) and STER were detected. The prevalence of STER was 100% of the samples, without significant differences among the four species; however, the prevalence and levels of this mycotoxin in the previously analyzed feed were low. This could be explained by the contamination of the farm environment. Animal biomonitoring can be a useful tool to assess animal exposure to mycotoxins. However, for these studies to be carried out and to be useful, knowledge must be increased on appropriate biomarkers for each mycotoxin in different animal species. In addition, adequate and validated analytical methods are needed, as well as knowledge of the relationships between the levels found in biological matrices and mycotoxin intake and toxicity.

## 1. Introduction

Food safety is of great importance worldwide, and it is a priority for the EU [1]. When human and animal food is evaluated for possible health risks, one of the aspects that should be considered is the presence of natural pollutants. These contaminants reach the food chain because they are naturally present in raw materials or due to the handling of crops, animals or foods [2]. Natural pollutants are mainly pesticides, preservatives, mycotoxins, or food additives, with mycotoxins being the group of main concern [3,4].

Mycotoxins are a large number of secondary metabolites synthesized by fungi growing on raw materials, particularly on cereals. They are characterized by a great diversity regarding their physicochemical characteristics. Moreover, they are generally not detected by organoleptic signals such as color or odor, and they are difficult to be eliminated during food processing due to their stability. More than a few of them have severe impacts on human and animal health, including, among others, hormonal disorders, neurotoxicity, nephrotoxicity, mutagenicity, effects on the immune system, gastrointestinal diseases, alterations in the growth of animals, diminished production of animal-derived products or lung problems in pigs [5,6,7,8,9,10].

Among mycotoxins, those of major concern are aflatoxins (AFs), produced by some species of *Aspergillus*, that usually appear in cereals, oilseeds, spices, and nuts; ochratoxin A (OTA), produced by *Aspergillus* and *Penicillium* fungi that appear in cereals, cereal-derived products, coffee, dried fruits or spices; trichothecenes, like deoxynivalenol (DON), nivalenol (NIV), T-2 and HT-2 toxins, and zearalenone (ZEA), produced by *Fusarium* fungi and also prevalent in cereals [5].

Compound feed (from now feed) for animals is mainly produced using cereals [11], and it has been proposed that crops could have a prevalence of mycotoxins between the 60–80%, especially in grains destined to feed preparation [12]. Through feeding, mycotoxins can reach animals, exerting their toxic effects such as those explained above. Also, they entail a high burden to farms and livestock due to animal death, diminished productivity, or the cost of treating symptoms caused by mycotoxin contamination [11]. Moreover, mycotoxins could be present in animal-derived products such as eggs, milk, or meat, reaching the human food chain [8,13,14].

It is known that one fungus species can produce different mycotoxins, and also, it is very probable that when fungal contamination is established on raw material, various fungi species be found. Then, the co-occurrence of various mycotoxins on the same product is the most likely scenario. Lauwers et al. [15] indicated that more than 70% of analyzed samples from feed presented levels of mycotoxins, and 38% of them presented co-occurrence.

To assess the exposure of animals to mycotoxins, the most widely used strategy is to analyze levels of these compounds in the feed and combine the results with feed intake data. In fact, the monitoring of the prevalence and levels of mycotoxins in the feed is carried out worldwide [16,17,18]. However, this approach has some drawbacks. Firstly, mycotoxins are not usually distributed homogenously on the raw material because fungal contamination can be present in limited zones. In this case, the sampling procedure is very important to obtain a representative sample [19]. Secondly, mycotoxins may be linked to or modified on the raw material, and then they are not detected in analytical methods previously prepared to quantify the corresponding parent compounds; so, contamination is underestimated. Finally, feed ingestion, and the processes of absorption, distribution, and elimination of mycotoxins, differ among individuals; therefore, the exposure of each individual is not evaluated [15,20].

Another approximation to solve these disadvantages is to evaluate the internal exposure of animals, that is: to quantify the presence of the parent or other related compounds in biological samples. The two approaches should not be seen as being in conflict but rather as complementary [21].

The chemical structures that, if measured in a biological matrix, can be correlated with exposure to mycotoxins are called biomarkers [22]. As an advantage, the biomonitoring of mycotoxins in an animal gives an idea of individual animal exposure not only through feed but also from other routes of contamination, such as inhalation or skin contact [20].

Due to the above reasons, Animal Biomonitoring (ABM) is a useful tool for assessing animal exposure to mycotoxins, in the same way as Human Biomonitoring (HBM) is for human exposure [23,24]. In fact, the use of this approach has grown in the latest years [25].

The most widely used technique for the quantification of mycotoxins in food, feed, and also in biological matrices has been liquid chromatography-tandem mass spectrometry (LC-MS/MS) [20]. This is due to its high sensitivity, necessary due to the low levels of biomarkers in biological matrices [25], and because it is able to simultaneously analyze compounds of different physicochemical properties such as mycotoxins; thus, the time employed for the quantification of several compounds and the cost of the analysis is affordable.

The objective of this study is to help increase knowledge about the exposure of food-producing animals to mycotoxins, in this case, in a region of northern Spain (Navarra). To this end, the biomonitoring of mycotoxins in cattle, poultry, pigs, and sheep plasma was carried out. The studied mycotoxins were selected due to their toxicity or prevalence: aflatoxin B1 (AFB1), OTA, ZEA, DON, and T-2 toxin, because their presence in animal plasma has not usually been studied: sterigmatocystin (STER), neosolaniol (NEO), diacetoxyscirpenol (DAS), fusarenon-X (FUS-X); and because of the chemical relationship with the former parent compounds: aflatoxin B2 (AFB2), G1 (AFG1), G2 (AFG2), and M1 (AFM1), 3-acetyldeoxynivalenol (3-ADON), 15-acetyldeoxynivalenol (15-ADON), ochratoxin B (OTB), NIV, deepoxy-deoxynivalenol (DOM-1), and HT-2 toxin.

Before plasma samples analysis, the revalidation of an analytical methodology previously developed and validated for the quantification of these 19 compounds in human plasma was carried out. This methodology has been applied to the biomonitoring of mycotoxins and their derivatives (including phase II metabolites: glucuronides and sulfates) in 80 animal plasma samples, 20 from each one of the animal species indicated above. Additionally, a comparison is made between the levels of mycotoxin in plasma and in the feed provided to the same animals.

## 2. Results

### 2.1. Analytical Method Revalidation

A previously validated method able to analyze the same mycotoxins as in the present study, but in human plasma [26], was revalidated for its use in animal plasma. The following parameters were assessed during revalidation: linearity has been proven in plasma from each one of the animal species, and good calibration curves were achieved in all cases because they accomplished the proposed criteria of acceptation (R^2^ > 0.99, relative error (RE)) in the percentage of back-calculated versus nominal concentration < 15% (20% at the limit of quantification (LOQ)). The linearity results for the matrix-matched calibration curves obtained for each mycotoxin in each animal species, without and with the enzymatic treatment of plasma, are shown in Table 1 and Table S1, respectively.

Moreover, the relationship between the qualification transition (q) and the quantification transition (Q) peak areas (q/Q) between a standard (fortified mobile phase) and a calibrator (fortified plasma) at the LOQ level has been calculated for pig plasma. For both parameters, a RE (%) < 20% has been obtained for all the mycotoxins (maximum value of 9.3% for AFG1). In the same way, the RE (%) for the retention times (RTs) between standards and calibrators were <2.5% (maximum obtained value of 1.5% for AFB2). Results are shown in Table S2.

Also, in pig plasma, matrix effect (ME) and recovery, at the LOQ level, and precision and accuracy at LOQ, 6× LOQ and 30× LOQ in whiting-day conditions, have been calculated for each mycotoxin. Results can be seen in Table 2 (ME, recovery, precision, and accuracy without enzymatic treatment) and S3 (ME, recovery, precision, and accuracy with enzymatic treatment).

ME and recovery values without (with) enzymatic treatment were between 82.9–116.4% (84.7–113.9%) and 81.2–108.9% (81.5–112.8%), respectively. Precision and accuracy were, for all levels and mycotoxins, <15%.

### 2.2. Analysis of the Samples

**Figure 1:**
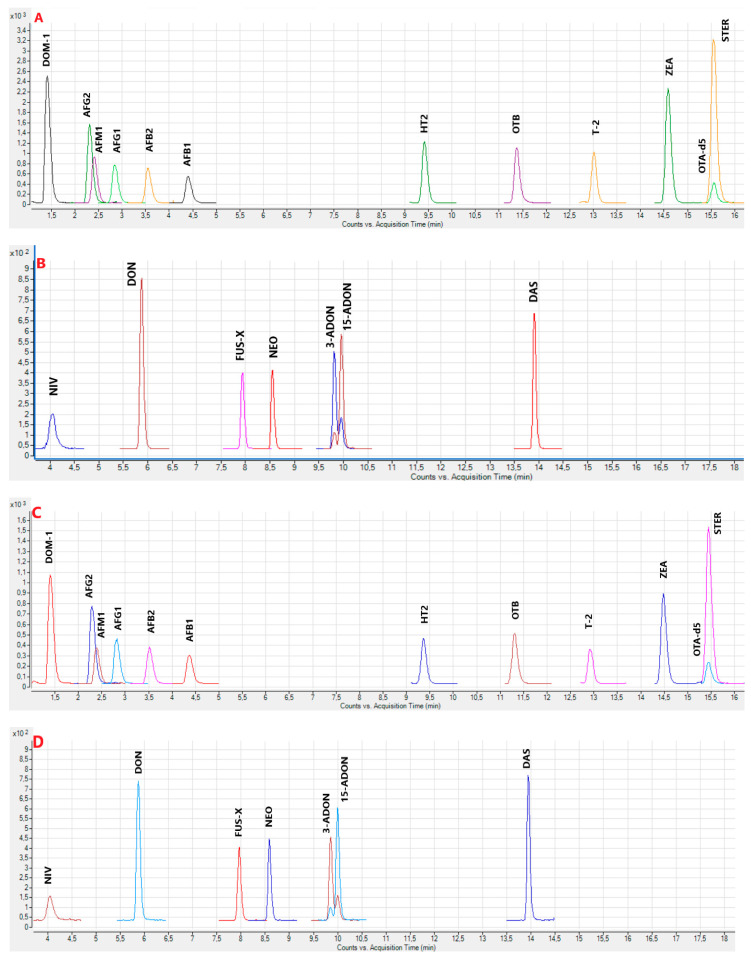

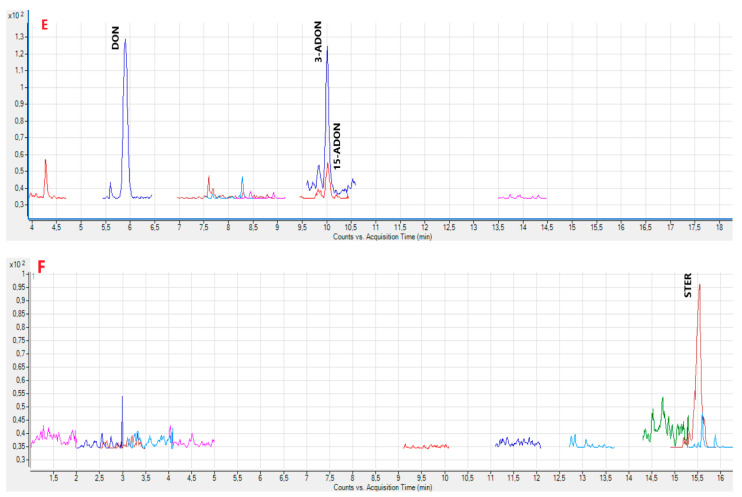
(**A**) Superposed chromatograms (Q) obtained from a calibrator at 10× LOQ level without enzymatic treatment (mycotoxins from group I). (**B**) Superposed chromatograms (Q) obtained from a calibrator at 10× LOQ levels without enzymatic treatment (mycotoxins from group II). (**C**) Superposed chromatograms (Q) obtained from a calibrator at 10× LOQ levels with enzymatic treatment (mycotoxins from group I). (**D**) Superposed chromatograms (Q) obtained from a calibrator at 10× LOQ levels with enzymatic treatment (mycotoxins from group II). (**E**) Superposed chromatograms (Q) obtained from a sample of poultry plasma without enzymatic treatment containing DON, 3- and 15-ADON). (**F**) Superposed chromatograms (Q) from a sample of poultry plasma with enzymatic treatment containing STER.

Figure 1A–F and Figure S1A–F show examples of the obtained chromatograms from calibrators and plasma samples, without and with enzymatic treatment, respectively.

In Table 3, the results obtained for RE (%) when comparing the q/Q ratio and the RT of the detected mycotoxins between calibrators and the samples can be seen.

In all cases, both transitions occurred in the chromatogram, and the RE (%) of the q/Q ratios, as well as of the RTs, were less than 20% and 2.5%, respectively. Therefore, the identity of the mycotoxins in the samples was confirmed.

Regarding the presence of mycotoxins without enzymatic treatment, no levels of the analyzed mycotoxins were found above the respective limit of detection (LOD). Only one sample of plasma from poultry contained quantifiable levels of DON and 3- and 15-ADON. DON presented a level of 13.2 ng/mL (LOQ: 8.7 ng/mL), while 3- and 15-ADON were at 2.3 and 2.7 ng/mL, respectively, close to the LOQ levels for these compounds.

With enzymatic treatment, the same trend has been observed; no mycotoxin levels have been detected. The only sample that presented DON and 3- and 15-ADON levels without the treatment with the mixture of β-glucuronidase-arylsulfatase, under enzymatic treatment, presented a slightly higher level for DON (14.0 ng/mL), but their derivatives were not detected.

The exception was STER, for which 100% of the plasma samples presented detectable levels. Eighty percent of the plasma samples from poultry and sheep had levels higher than their respective LOQs, whereas, for cattle and pigs, this percentage was higher in both cases (95%). Results obtained for STER (ng/mL) in each sample under enzymatic treatment are in Table S4, whereas a summary of these results is shown in Table 4.

No significant differences have been found in the levels of STER among the animal species (*p* = 0.1313 in the Kruskal Wallis test).

## 3. Discussion

The exposure to mycotoxins of animals destined for the production of human food is a global health and economic problem with no easy solutions. Good agricultural practices during the pre- and post-harvest periods could help decrease fungal growth on grains and raw materials, but other factors, such as weather conditions or the globalization of the commodity markets, are not easy to control. In addition, once the raw material is contaminated, the total elimination of mycotoxins is not possible [27]. Therefore, it is necessary to improve the knowledge and control of the exposure to mycotoxins of those animals.

The main objective of this study is to contribute to these needs. For this, and to know the animal exposure, the second approach has been chosen; that is, to evaluate the internal exposure of the animals through the analysis of biomarkers in plasma.

Arce-Lopez et al. [20] reported what HBM requires: validated analytical methods, a selection of suitable biomarkers, and accessible biological matrixes, such as plasma. Thus, the same requirements should be needed for ABM.

Validated analytical methods, capable of simultaneously determining several mycotoxins and their derivatives, are needed not only for exposure control but also for carrying out metabolism or toxicokinetics studies. To the best of our knowledge, very few have been developed for animal plasma samples. For instance: Devreese et al. [28] developed and validated a multimycotoxin analytical method for DON, DOM-1, T-2, HT-2, ZEA and its derivatives in pig plasma. De Baere et al. for FB1, its hydrolyzed metabolites and FB2 in chicken plasma [29], for AFs in plasma from chickens and cattle [30], and also for T-2, HT-2, DON, and DOM-1 in plasma samples from pigs and chickens [31]. Lauwers et al. [15] developed a methodology to determine 24 mycotoxins in plasma from pig and chicken: AFB1, AFM1, OTA, FB1, T-2, HT-2, ZEA (and 5 derivatives), DON, DOM-1, 3- and 15-ADON, enniatins (A, A1, B, B1), beauvericin, alternariol, alternariol-monomethyl ether, and tenuazonic acid. Broekaert et al. [32] achieved the quantification of 3- and 15-ADON, DON and DOM-1 in chicken and pig plasma. Of them, only a few were able to simultaneously determine mycotoxins from different families [15,28,31].

Due to the need for multimycotoxin analytical methods for use in animal plasma, in the present study, a methodology capable of quantifying 19 compounds (mycotoxins and their derivatives) in human plasma [26] has been revalidated for the determination of these toxins in plasma samples from four different food-producing animal species (cattle, pigs, poultry and sheep). Among them, mycotoxins previously analyzed in the literature in animal plasma (although only from pigs or chicken) such as AFB1, OTA, ZEA, DON, 3- and 15-ADON, DOM-1, T-2, HT-2, and others not usually studied in animal plasma, such as AFM1, STER, NEO, DAS, FUS-X, AFB2, AFG1, AFG2, OTB, and NIV. Likewise, the analysis of plasma samples treated with a mixture of β-glucuronidase-arylsulfatase provided information on possible glucuronide or sulfate conjugates.

There are no validation guidelines for analytical methods for mycotoxin determination in animal biological fluids. Then, some European-related documents have been consulted regarding the performance of analytical methods and the interpretation of results (2002/657/EC) [33], and the bioanalytical method validation (European Medicines Agency (EMA) guideline) [34]. A complete validation has not been carried out because the same matrix (plasma) has been used, although from different species [34].

In summary, to quantify mycotoxins in plasma samples, matrix-matched calibrators have been prepared using plasma from each of the animal species. The obtained calibration curves complied with the criteria indicated in the EMA bioanalytical method validation guideline [34]. Also, the identity of mycotoxins in the samples was assessed by comparing the mycotoxin RT and q/Q relationship between the calibrators and the samples. The obtained LOD values were in the ng/mL range (see material and methods section). They have been considered low enough and adequate for mycotoxin determination in animal plasma, as indicated by other authors [28].

Regarding biomarkers, it is essential to select the appropriate molecule and biological matrix in which to be analyzed so that the levels obtained can be related to the exposure to each parent compound. However, very few have been identified [35]. Firstly, because the metabolism of mycotoxins is not well known and many more toxicokinetic studies in animals are needed [25]. In the few toxicokinetic studies carried out, mainly in pigs or chickens, some mycotoxin biomarkers in animal plasma are suggested. In most cases, the biomarker analyzed is the parent compound, for example, DON [25], OTA and AFB1 [15,36] or T-2 [25], whereas in other cases, the biomarker chosen is a metabolite of the parent compound, for example, DOM-1 [25], OTB and OTα [35], HT-2 toxin, and T-2 triol [25]. Also, phase II metabolites, especially glucuronides or sulfates of the parent compound, are proposed [15,25]. In the present study, the chosen biomarkers included parent compounds and some of their metabolites; moreover, an enzymatic treatment has been used in order to detect the mycotoxins that appear in their conjugated forms.

In addition, the presence of mycotoxins in animal plasma was compared with the data obtained in a recently published paper [37] in which four hundred feed samples intended for feeding these animal species (100 for each one) were collected from farms in Navarra (a region of Northern Spain). Plasma samples for the present study were obtained from the same farms and simultaneously with the collection of feed samples. Feed samples were analyzed for AFB1, AFB2, AFG1, AFG2, ZEA, OTA, OTB, DON, and STER. The results of that study indicated that one or more of these mycotoxins appeared in all types of feed (for cattle, pigs, poultry, and sheep) (63.5% of the samples presented 2 to 5 mycotoxins). ZEA and DON presented the highest prevalence in all types of feed. In general, the levels found for the mycotoxins regulated in the EU in feed (OTA, DON, ZEA, and AFB1) were below what was established. Mean and median values for all mycotoxins and in all feed types were <LOD (<LOQ for STER), except for ZEA (mean values: 65.4–104.7 µg/kg) and DON (mean values 113.4–176.0 µg/kg) but both mycotoxins were below the maximum levels regulated by the EU.

In the animal plasma samples analyzed without the enzymatic treatment, no mycotoxins previously found in the feed (AFs, OTA, ZEA, DON, or STER), although at low levels, were detected. Only one sample from poultry presented levels of DON (13.2 ng/mL) and its derivatives 3- and 15-ADON. Den Hollander et al. [19] studied the relationship between DON levels in feed and in broiler chicken serum. In this case, the DON levels in the feed were quite similar to those obtained in the feed for poultry in the study of Muñoz-Solano and González-Peñas [37]: prevalence of 75% and 71%, respectively; mean values 270 µg/kg and 176 µg/kg (255.3 µg/kg mean of positive samples), respectively; and maximum value of 751 and 755 µg/kg, respectively. And also, the results in serum were quite similar to those of the present study: Den Hollander et al. [19] found a prevalence of DON much less than in feed (17.5%) and a mean value of 11.7 ng/mL.

A possible explanation for the no detection of DON could be that, in general, DON is rapidly absorbed by animals, with species-dependent bioavailability: low in chickens and sheep and higher in pigs [38,39]. However, once DON is absorbed, it is rapidly distributed to different organs, and the levels in plasma decline quickly [19].

DOM-1 was not detected in any plasma sample. It has been reported that this compound was only found in pig serum when the feed was highly contaminated with DON (>2000 µg/kg feed) [25]. This agrees with the results obtained in the present study because de maximum level of DON found in the feed was 887 µg/kg [37].

After the enzymatic treatment, the sample previously containing DON presented a level slightly higher than before, and its derivatives were not detected. Poultry metabolize DON through sulfate conjugation, whereas pigs use glucuronidation [15,38]. Since a mixture of β-glucuronidase-arylsulfatase was used in the present study, both conjugates could be converted to the parent compound and thus quantified. Dänicke et al. [40] reported the increase of DON concentration in pig serum after incubation with β-glucuronidase since DON appears as conjugated in approximately 33% of this animal species.

An interesting fact is the detection of STER in 100% of the samples, but only with enzymatic treatment. This agrees with Fushimi et al. [41], which indicated that STER is extensively conjugated with glucuronic acid in the liver of cattle. However, in the analyzed feed, only 7% of the samples contained detectable levels of this mycotoxin at levels between <LOD to 6.1 µg/kg [37].

A possible explanation for these results is that STER, structurally similar to AFB1 and a precursor in its biosynthetic pathway, appears in grains and grain-based products infested by *A. versicolor*. This fungus cannot transform STER into AFB1, and therefore, in this case, STER appears as a contaminant instead of AFs. On the contrary, raw materials infested by *A. flavus* and/or *A. parasiticus* mostly contain AFs levels because STER has been converted into these compounds [42]. In pig production farms, *A. versicolor* was found to be the most prevalent species both in the air and on surfaces. Since mycotoxins are not volatile, their presence in the air could be due to aerosols formed during routine animal and farm handling [43].

Therefore, the presence of STER and DON in the plasma samples can be explained not only by the presence in the feed used for the animals, in which low levels were found, especially of STER, but also because the farm environment plays its role in the exposure of animals to mycotoxins. Viegas et al. [43] indicated that the farm environment is likely to promote mycotoxin production and reported that STER was detected in all litter analyzed samples at values up to 2.69 ng/g, as well as DON at levels up to 76.4 ng/g.

STER is known to be hepatotoxic in poultry and pigs and nephrotoxic in poultry, but there are no data on the exposure in food-producing animals; therefore, the risk to animal health cannot be established [42]. Therefore, and due to its toxicity, further studies are needed to obtain these data and to determine sources, other than feed, for animal exposure.

## 4. Conclusions

In the present study, the exposure of animals from Navarra (northern Spain) to mycotoxins has been assessed through animal biomonitoring. Firstly, a methodology capable of analyzing 19 compounds (AFB1, OTA, ZEA, DON, 3- and 15-ADON, DOM-1, T-2, HT-2, AFM1, STER, NEO, DAS, FUS-X, AFB2, AFG1, AFG2, OTB, and NIV) in human plasma was successfully revalidated to be used in animal plasma. Then, these compounds were analyzed in 80 plasma samples obtained from animals dedicated to food production: cattle, pigs, poultry, and sheep. In order to determine some possible phase II metabolites (glucuronides and sulfates), samples have been analyzed without and with a treatment with a mixture of β-glucuronidase-arylsulfatase.

No mycotoxin or their derivatives were detected in any of the samples without enzymatic treatment, so the exposure of these animals to mycotoxins is low. Only one sample from poultry presented quantifiable levels of DON and 3- and 15-ADON. This result agrees with the low levels obtained in the feed used to feed these animals, in which the regulated mycotoxins were below the maximum levels established in the EU.

After enzymatic treatment, STER was detected in 100% of the samples, and no significant differences have been found among the four species, although the prevalence and levels of this mycotoxin in the previously analyzed feed were low. The exposure of animals to this mycotoxin could be explained by contamination of the farm environment. Due to the toxic effects of STER, further studies are needed to determine sources, other than feed, for animal exposure.

ABM can be a useful tool to assess animal exposure to mycotoxins, as is HBM for human exposure. However, for these studies to be carried out and to be useful, knowledge must be increased, based on toxicokinetic and metabolic studies, on appropriate biomarkers for each mycotoxin in different biological matrices and animal species. In addition, validated analytical methods are needed for the analysis of multiple mycotoxins in different biological matrices, as well as knowledge of the relationships between levels found in biological matrices and mycotoxin intake and toxicity.

## 5. Materials and Methods

### 5.1. Chemical Reagents and Materials

The reagents used were: acetonitrile (ACN) and methanol (MeOH) (LC/MS grade) from Scharlab (Barcelona, Spain); formic acid (MS grade, purity >98%), β-glucuronidase-arylsufatase (from Helix Pomatia), and ammonium formate (MS grade) from Merck (Darmstadt, Germany); and SPE Captiva EMR-lipid of 3 mL from Agilent Technologies (Santa Clara, CA, USA). Type I water was obtained from water purifying equipment from Wasserlab (Barbatáin, Spain).

### 5.2. Mycotoxin Standards

AFB1 2 μg/mL (PubChem CID: 186907), AFB2 0.5 μg/mL (PubChem CID: 2724360), AFG1 2 μg/mL (PubChem CID: 14421), AFG2 0.5 μg/mL (PubChem CID: 2724362), AFM1 0.5 μg/mL (PubChem CID: 15558498), Ochratoxin A-(phenyl-d5) (OTA-d5) 10 µg/mL (PubChem CID: 71751270), OTB 10 µg/mL (PubChem CID: 20966), and ZEA 100 µg/mL (PubChem CID: 5281576), STER 50 μg/mL (PubChem CID: 5280389), DOM-150 μg/mL (PubChem CID: 119324), NIV 100 μg/mL (PubChem CID: 5284433), DON 100 μg/mL (PubChem CID: 40024), 3-ADON 100 μg/mL (PubChem CID: 5458510), 15-ADON 100 μg/mL (PubChem CID: 10382483), NEO 100 μg/mL (PubChem CID: 13818797), DAS 100 μg/mL (PubChem CID: 15571694), FUS-X 100 μg/mL (PubChem CID: 304599), T-2 100 μg/mL (PubChem CID: 5284461), HT-2 100 μg/mL (PubChem CID: 10093830) were obtained from Sigma-Aldrich (Merck KGaA, Darmstadt, Germany). All mycotoxins (with a purity of more than 98%) were purchased as an ACN solution.

### 5.3. Safety Precautions

Mycotoxins are compounds that are toxic to humans. In order to minimize the risk of exposure in the laboratory, some safety precautions were considered. Mycotoxin standards were always handled in solution. Also, gloves, protective masks, and a laminar flow hood during the handling of samples or mycotoxin solutions were used as personal protection measures. Finally, the work was carried out in low light conditions to prevent the degradation of photosensible mycotoxins.

### 5.4. Animal Plasma Samples

Samples were collected between 2019 and 2020 from different farms in Navarra (northern Spain). A total of 20 animals of each one of the 4 species (cattle, pigs, poultry, and sheep) were employed. Blood was obtained from each one of the animals by the corresponding veterinary staff during the routine health control in the farms, and no extra extraction was carried out for this study. Ten mL of the total blood extracted was separated and collected in 10 mL BD vacutainer^®^ plasma tubes (Madrid, Spain) with EDTA as an anticoagulant. Once the samples were in the laboratory, plasma was obtained by centrifuging the sample at 12,000× *g* for 10 min at 4 °C. The resulting plasma was stored deep-frozen at −80 °C until analysis.

### 5.5. Preparation of Mycotoxin Solutions and Calibrators

The appropriate volume of each one of the individual mycotoxin solutions was mixed to prepare a stock solution containing all mycotoxins in ACN at the concentrations shown in Table 5. OTA-d5 was used instead of OTA in the preparation of calibrators. This is due to the fact that, in a previous research work using human plasma [26], no OTA-free plasma was found that could be used to prepare calibrators; so, before starting the present study, it was decided to follow the same strategy.

Matrix-matched calibrators were prepared using plasma obtained from the different species. Plasma was fortified as follows: the appropriate volume from the stock solution was taken and put into a 15 mL tube, then this volume was dried in a vacuum centrifugal evaporator at 60 °C (Genevac™ miVac Centrifugal Concentrator) from Fisher Scientific (Barcelona, Spain). Afterward, 15 µL of ACN was added to the tube, and it was vortexed for 5 min to completely redissolve the mycotoxins. Subsequently, 485 µL of animal plasma was added and vortexed again for 5 min. Finally, each of the matrix-matched calibration points was submitted to the sample preparation procedure prior to chromatographic analysis.

In the preparation of calibrators to analyze samples with enzymatic treatment, plasma was fortified, as explained above, and then treated with the mixture of β-glucuronidase-arylsulfatase, as indicated below.

With this procedure, 8 matrix-matched calibration points (1, 2, 4, 6, 8, 10, 20, and 30× LOQ) were prepared using plasma for each one of the studied species, and without and with enzymatic treatment. The LODs, LOQs, and calibration ranges of mycotoxins in plasma are shown in Table 6.

### 5.6. Sample Preparation

Plasma samples were prepared following the procedure described by Arce-López et al. [26]. Briefly: 400 mL of plasma was added to the SPE Captiva EMR lipid cartridge containing 1.2 mL of acidified ACN (1% formic acid) using a vacuum manifold. The eluate was separated into 2 different 15 mL polypropylene tubes (0.4 mL each), and both were evaporated to dryness in a centrifugal vacuum evaporator at 60 °C. One of them (intended for the analysis of mycotoxins classified as Group I) was reconstituted with 200 µL of 60% A/40% B mobile phase (A: 5 mM ammonium formate and 0.1% formic acid in water; B: 5 mM ammonium formate and 0.1% formic acid in a 95:5 MeOH/water (*v*/*v*)). The second tube (intended for the analysis of mycotoxins classified as Group II) was also reconstituted with 200 µL of mobile phase, but in relation to 95% A/5% B. Finally, the obtained solutions were vortexed and filtered through a 0.45 µm PVDF filter (Merck, Millipore, Ireland) before injection.

Moreover, to quantify possible phase II metabolites (glucuronide and sulfate conjugates), another aliquot of each animal plasma sample was treated with a mixture of β-glucuronidase-arylsulfatase. For this, 50 µL of the enzyme solution was added to 450 µL of animal plasma and incubated overnight at 37 °C. The next day the sample processing continued as described above.

### 5.7. Analysis of Samples

Chromatographic separation and quantification were carried out in an Agilent Technologies 1200 series LC-MS/MS, connected to an Agilent Technologies (USA) triple quadrupole mass detector (6410 b) in ESI(+) mode. The acquisition parameters (Fragmentor voltage, collision energy and transitions) are described in Flores–Flores et al. [44,45]. For OTA-d5, the detection parameters were those for OTA, except for the used transitions (Q: 409.1–239.0; q: 409.1–102.1) [26].

The column used was a 150 mm × 2.1 mm × 2.7 µm Ascentis express C18 with solid core from Merck (Darmstadt, Germany). The separation temperature was 45 °C, and the injection volume was 20 µL.

The 19 mycotoxins were divided into 2 groups (I and II) based on the different compositions of the initial mobile phase and also on the different elution gradients they needed. The composition of the solutions for the mobile phase was common for both groups: A was 5 mM ammonium formate and 0.1% formic acid in water, while B was 5 mM ammonium formate and 0.1% formic acid in a 95:5 MeOH/water (*v*/*v*).

The gradient used in the analysis of Group I mycotoxins (DOM-I, AFG2, AFG1, AFB2, AFB1, AFM1, OTB, OTA-d5, ZEA, T2, HT2, STER) was as follows: 0 min 40% B to 72% B at 20 min. Then, at 20.1 min, they returned to the initial mobile phase (40% B), and these conditions were maintained for 10 min (post-time).

In the analysis of Group II mycotoxins (NIV, NEO, DON, FUS-X, 3-ADON, 15-ADON, DAS), the gradient started at 5% B, to 28% B at 5 min, to 45% at 10.5 min, to 60% B at 11.0 min, to 90% B at 15.5, to 5% B at 16.0 min, then 10 min of post-time.

All samples were analyzed in batches, one for each animal species, which included matrix-matched calibrators and samples. Mycotoxins in samples were quantified using the corresponding calibration curve prepared in each batch.

### 5.8. Method Revalidation

Linearity was assessed using matrix-matched calibrators for each one of the studied species. Eight points were prepared for making the calibration curves. Acceptance criteria were: R^2^ > 0.99, RE (in percentage) of back-calculated versus nominal concentration < 15% (20% at LOQ) in a minimum of 6 calibrators [34]. Identification of each mycotoxin was based on the presence of the quantification and qualification transitions and the coincidence of RTs between a standard (fortified mobile phase) and a calibrator (fortified plasma) analyzed in the same batch. For each one of the mycotoxins, the ratio q/Q was obtained in the standard, and this value was compared with that obtained in the calibrator at the LOQ level. These 2 values should not differ by more than 20% using the following equation:

$$\mathrm{RE}\;\left(\%\right)=\left(\frac{\frac{q}{Q}\;\mathrm{standard}-\frac{q}{Q}\;\mathrm{calibrator}}{\frac{q}{Q}\;\mathrm{standard}}\right)\;\times \;100$$

In addition, RTs for each mycotoxin in the calibrator should not differ from that in the standard by more than 2.5% [33].

Also, accuracy and precision were studied at LOQ, 6× LOQ y 30× LOQ in within-day conditions (3 matrix-matched calibrators per concentration level in 1 day). The acceptance criteria for both parameters were <15% (20% at LOQ) [34].

ME and recovery were also studied at the LOQ level using the following equations:

$$\mathrm{ME}\;\left(\%\right)=\frac{\mathrm{area}\left(\mathrm{spiked}\;\mathrm{plasma}\right)}{\mathrm{area}\left(\mathrm{standard}\right)}\;\times \;100$$

$$\mathrm{Recovery}\;\left(\%\right)=\frac{\mathrm{area}\left(\mathrm{spiked}\;\mathrm{plasma}\right)}{\mathrm{area}\left(\mathrm{spiked}\;\mathrm{extract}\right)}\;\times \;100$$

Except for linearity, all these parameters were assessed using only pig plasma due to the low available volume in the case of the other species.

### 5.9. Identification of Mycotoxins in Samples

In the same way as in revalidation, the identification of each mycotoxin in each sample was based on the presence of the quantification and qualification transitions and the coincidence of RTs in calibrators and in samples. For each one of the mycotoxins, the mean ratio q/Q and mean RT values were obtained for calibrators, and these values were compared with those obtained in each sample. These 2 values should not differ by more than 20% (q/Q) and 2.5% (RT), respectively, using the following equation.

$$\mathrm{RE}\;\left(\%\right)=\left(\frac{\mathrm{Mean}\;\mathrm{value}\;\mathrm{in}\;\mathrm{calibrators}\;\left(\mathrm{ME}\;\mathrm{or}\;\mathrm{RT}\right)-\mathrm{value}\;\mathrm{in}\;\mathrm{sample}\;\left(\mathrm{ME}\;\mathrm{or}\;\mathrm{RT}\right)}{\mathrm{Mean}\;\mathrm{value}\;\mathrm{in}\;\mathrm{calibrators}\;(\mathrm{ME}\;\mathrm{or}\;\mathrm{RT})}\right)\times \;100$$

### 5.10. Statistical Analysis

The STATA/IC 12.0 program was used for statistical analysis in order to compare STER levels between different animal species. Since the data did not have a normal distribution, a Kruskal–Wallis test was used. For statistical significance, a probability value of 0.05 was defined.

## Figures and Tables

**Table 1:** Linearity results for the matrix-matched calibration curves obtained for each mycotoxin in plasma without enzymatic treatment.

Mycotoxin	Cattle			Pigs			Poultry			Sheep		
	R^2^	Equation	RE * (%)	R^2^	Equation	RE (%)	R^2^	Equation	RE (%)	R^2^	Equation	RE (%)
DOM-1	0.9983	y = 15.86x − 31.47	<14.6	0.9989	y = 16.76x − 25.10	<13.1	0.9950	y = 15.47x − 26.37	<16.0	0.9997	y = 15.69x − 24.00	<19.6
AFG2	0.9987	y = 146.91x − 11.44	<16.1	0.9983	y = 152.77x − 51.19	<19.4	0.9970	y = 150.42x − 19.02	<10.3	0.9992	y = 145.06x + 4.12	<16.8
AFM1	0.9992	y = 108.30x + 5.20	<13.0	0.9983	y = 143.67x + 15.50	<15.4	0.9995	y = 146.32x − 13.84	<19.4	0.9955	y = 145.44x − 15.21	<15.8
AFG1	0.9996	y = 324.75x − 36.60	<19.6	0.9956	y = 401.38x − 41.55	<17.0	0.9987	y = 394.98x + 13.83	<16.2	0.9973	y = 395.46x − 12.15	<16.1
AFB2	0.9993	y = 415.65x − 21.48	<11.8	0.9982	y = 373.74x + 12.14	<16.9	0.9976	y = 389.17x − 10.79	<12.6	0.9990	y = 374.46x − 45.24	<11.8
AFB1	0.9994	y = 561.42x + 2.56	<19.9	0.9991	y = 603.48x − 59.23	<16.1	0.9987	y = 560.27x + 18.73	<18.6	0.9978	y = 592.19x − 0.10	<16.9
HT-2	0.9977	y = 20.19x − 37.72	<14.8	0.9991	y = 17.81x + 38.29	<18.4	0.9957	y = 18.94x + 22.20	<12.5	0.9989	y = 19.06x − 29.50	<17.8
OTB	0.9965	y = 112.87x + 7.69	<15.4	0.9977	y = 122.41x − 19.96	<14.0	0.9973	y = 109.23x + 2.71	<14.3	0.9979	y = 118.62x − 16.35	<12.9
T-2	0.9990	y = 100.99x + 3.81	<18.0	0.9987	y = 113.49x + 21.60	<17.3	0.9986	y = 120.12x − 21.19	<14.3	0.9967	y = 111.35x − 11.23	<12.7
ZEA	0.9990	y = 25.43x − 38.81	<16.7	0.9987	y = 28.71x − 39.60	<16.2	0.9961	y = 26.63x + 2.15	<13.8	0.9969	y = 29.68x + 8.34	<13.1
OTA-d_5_	0.9996	y = 74.91x − 52.75	<18.8	0.9968	y = 56.95x + 1.14	<13.0	0.9973	y = 62.52x − 18.50	<10.4	0.9937	y = 58.67x − 31.63	<13.7
STER	0.9998	y = 191.70x − 65.30	<16.9	0.9959	y = 111.19x + 50.23	<19.5	0.9936	y = 120.34x − 35.22	<14.5	0.9983	y = 123.31x − 35.46	<19.4
NIV	0.9978	y = 3.67x − 9.42	<15.7	0.9967	y = 3.63x + 13.43	<13.8	0.9968	y = 3.41x + 24.66	<18.6	0.9970	y = 3.80x − 10.51	<11.4
DON	0.9960	y = 8.04x + 21.82	<18.4	0.9971	y = 8.78x − 9.97	<11.7	0.9997	y = 8.78x − 16.46	<8.8	0.9988	y = 9.38x − 30.31	<12.5
FUS-X	0.9990	y = 11.59x − 29.51	<15.3	0.9941	y = 11.54x − 55.12	<18.6	0.9967	y = 11.32x − 14.72	<5.7	0.9906	y = 10.75x + 1.42	<19.1
NEO	0.9957	y = 99.31x − 11.48	<14.3	0.9989	y = 90.43x + 12.09	<12.8	0.9938	y = 94.17x + 7.16	<11.5	0.9934	y = 103.78x − 24.38	<8.9
3-ADON	0.9918	y = 44.18x − 19.72	<15.2	0.9946	y = 42.82x − 6.24	<16.5	0.9979	y = 46.71x − 13.94	<17.5	0.9934	y = 46.65x − 8.59	<11.8
15-ADON	0.9971	y = 30.49x − 34.99	<13.6	0.9984	y = 28.24x − 31.74	<10.5	0.9961	y = 27.39x − 0.51	<14.2	0.9945	y = 29.33x − 10.39	<10.7
DAS	0.9941	y = 107.62x − 6.98	<14.1	0.9988	y = 111.37x − 13.16	<8.0	0.9990	y = 107.56x − 19.55	<17.6	0.9986	y = 116.46x − 7.33	<16.0

* RE (%): The maximum value of relative errors of back-calculated concentrations is shown.

**Table 2:** Matrix effect (%) and recovery (%), both at LOQ; precision (RSD%), and accuracy (RE%) in fortified pig plasma and without enzymatic treatment.

Mycotoxin	ME (%)	Recovery (%)	Precision (RSD%) (n = 3)			Accuracy (RE%) (n = 3)		
			LOQ	6× LOQ	30× LOQ	LOQ	6× LOQ	30× LOQ
DOM-1	93.2	97.0	5.4	4.1	2.7	5.8	1.9	1.1
AFG2	98.8	90.7	5.5	6.3	1.8	12.7	3.3	0.9
AFM1	103.8	91.8	9.6	2.6	1.7	5.6	2.1	1.1
AFG1	107.7	88.6	4.5	4.0	1.4	15.7	4.0	2.0
AFB2	93.5	100.3	6.3	3.0	2.0	10.6	2.6	1.6
AFB1	99.7	94.0	4.3	2.8	1.3	12.7	8.4	0.4
HT-2	94.5	95.8	13.2	4.9	2.9	4.0	2.0	1.0
OTB	92.3	96.9	9.7	4.9	1.9	2.7	3.8	1.3
T-2	93.0	90.9	7.9	2.8	1.4	9.3	2.3	1.1
ZEA	110.7	96.3	7.7	5.8	2.2	7.1	0.4	0.7
OTA-d_5_	89.8	83.7	11.0	4.2	3.3	1.4	8.0	2.0
STER	91.7	81.2	8.8	2.6	2.1	10.0	1.3	3.0
NIV	107.5	92.5	9.7	1.9	3.8	18.9	5.0	2.5
DON	92.5	86.8	9.2	4.9	5.5	8.8	6.8	2.5
FUS-X	108.2	104.0	3.2	4.2	5.1	14.4	2.9	2.0
NEO	83.4	102.9	11.6	4.3	2.6	7.4	3.2	0.5
3-ADON	116.4	95.5	7.0	3.3	3.2	9.8	9.5	0.8
15-ADON	96.2	108.9	6.5	6.6	4.4	13.3	1.0	1.0
DAS	82.9	102.6	4.3	4.4	2.2	10.5	3.5	1.8

LOQ: limit of quantification. RSD: relative standard deviation, RE: relative error.

**Table 3:** Values of RE (%) when comparing q/Q ratio and retention times (RT) of the detected mycotoxins between calibrators and samples.

Mycotoxin	Mean q/Q Ratio Calibrators (%)	Mean q/Q Ratio Samples (%)	RE(%)	Mean RT Calibrators (min)	Mean RT Samples (min)	RE(%)
Poultry (without enzymatic treatment) (sample identification: 20)						
DON	90.43	93.27	3.0	2.38	2.36	0.8
3-ADON	76.97	75.25	2.2	9.92	9.92	0.1
15-ADON	91.14	87.95	3.5	10.11	10.12	0.1
Poultry (with enzymatic treatment) (sample identification: 20)						
DON	91.22	85.13	6.7	2.38	2.37	0.4
STER	86.50	88.31	2.0	15.75	15.74	0.1
Pigs (with enzymatic treatment)						
STER	90.15	86.94	3.6	15.74	15.74	0.0
Cattle (with enzymatic treatment)						
STER	87.00	88.17	1.3	15.75	15.74	0.1
Sheep (with enzymatic treatment)						
STER	84.69	88.25	4.0	15.74	15.74	0.0

**Table 4:** Results obtained for STER in samples with enzymatic treatment of the plasma.

Parameter	Cattle	Poultry	Pigs	Sheep
% Positive samples (>LOD)	100	100	100	100
% Positive samples (>LOQ)	95	80	95	80
Mean value of positive samples (>LOD) (ng/mL)	2.4	2.0	2.9	2.4
Median value (ng/mL)	2.0	1.7	2.6	2.3
1st Quartile (ng/mL)	1.4	1.1	1.8	1.1
3rd Quartile (ng/mL)	3.5	2.8	4.4	3.6
Maximum level found (ng/mL)	4.3	3.9	5.0	4.9

LOD for STER: 0.20 ng/mL. LOQ: 1 ng/mL.

**Table 5:** Mycotoxin concentration in the stock solution.

Group I				Group II	
Mycotoxin	ng/mL	Mycotoxin	ng/mL	Mycotoxin	ng/mL
DOM-1	300	T2	50	NIV	1020
AFG2	40	ZEA	200	DON	435
AFM1	40	OTA-d5	100	Fus-X	350
AFG1	15	STER	50	NEO	40
AFB2	15	HT2	300	3-ADON	87
AFB1	10			15-ADON	136
OTB	50			DAS	35

**Table 6:** Limits of quantification, detection, and calibration range of mycotoxins in plasma.

Mycotoxin	LOD (ng/mL)	LOQ (ng/mL)	Calibration Range (ng/mL)
AFG2	0.35	0.8	0.8–24.0
AFG1	0.07	0.3	0.3–9.0
AFB2	0.07	0.3	0.3–9.0
AFB1	0.04	0.2	0.2–6.0
AFM1	0.18	0.8	0.8–24.0
DOM-1	1.35	6.0	6.0–180.0
3-ADON	0.70	1.8	1.8–52.5
15-ADON	1.20	2.7	2.7–81.6
T-2	0.20	1.0	1.0–30.0
HT-2	2.70	6.0	6.0–180.0
DAS	0.15	0.7	0.7–21.0
NEO	0.18	0.8	0.8–24.0
FUS-X	1.95	8.7	8.7–261.6
OTA-d5	0.40	2.0	2.0–60.0
OTB	0.40	1.0	1.0–30.0
ZEA	1.80	4.0	4.0–120.0
STER	0.20	1.0	1.0–30.0
NIV	9.10	20.4	20.4–612.0
DON	1.94	8.7	8.72–261.6

LOQ: limit of quantification. LOD: limit of detection.

## Data Availability

Not applicable.

## Supplementary Materials

The following supporting information can be downloaded at: https://www.mdpi.com/article/10.3390/toxins15040295/s1: Table S1: Linearity results for the matrix-matched calibration curves obtained for each mycotoxin in plasma with enzymatic treatment; Table S2: Qualification/quantification peak areas ratio and retention times (min) for each one of the mycotoxins in a standard (fortified mobile phase) and a calibrator (fortified pig plasma) at LOQ level without enzymatic treatment; Table S3: Matrix effect and recovery (at LOQ), and precision, and accuracy in fortified pig plasma and with enzymatic treatment; Table S4: Results obtained for STER (ng/mL) in samples of enzymatically-treated plasma. Figure S1: (A) Superposed chromatograms (q) obtained from a calibrator at 10× LOQ level without enzymatic treatment (mycotoxins from group I). (B) Superposed chromatograms (q) obtained from a calibrator at 10× LOQ levels without enzymatic treatment (mycotoxins from group II). (C) Superposed chromatograms (q) obtained from a calibrator at 10× LOQ levels with enzymatic treatment (mycotoxins from group I). (D) Superposed chromatograms (q) obtained from a calibrator at 10× LOQ levels with enzymatic treatment (mycotoxins from group II). (E) Superposed chromatograms (q) obtained from a sample of poultry plasma without enzymatic treatment containing DON, 3- and 15- ADON). (F) Superposed chromatograms (q) from a sample of poultry plasma with enzymatic treatment containing STER.

## Abbreviations

15-ADON	15-acetyldeoxynivalenol
3-ADON	3-acetyldeoxynivalenol
ABM	Animal biomonitoring
ACN	Acetonitrile
AFB1	Aflatoxin B1
AFB2	Aflatoxin B2
AFG1	Aflatoxin G1
AFG2	Aflatoxin G2
AFM1	Aflatoxin M1
AFs	Aflatoxins
DAS	Diacetoxyscirpenol
DOM-1	Deepoxy-deoxynivalenol
DON	Deoxynivalenol
EMA	European Medicines Agency
FUS-X	Fusarenon-X
HBM	Human biomonitoring
LC-MS/MS	Liquid chromatography-tandem mass spectrometry
LOD	Limit of detection
LOQ	Limit of quantification
ME	Matrix effect
MeOH	Methanol
NEO	Neosolaniol
NIV	Nivalenol
OTA	Ochratoxin A
OTA-d_5_	Ochratoxin A-(phenyl-d_5_)
OTB	Ochratoxin B
q	Transition of qualification
Q	Transition of quantification
RE	Relative error
RT	Retention Time
STER	Sterigmatocystin
ZEA	Zearalenone
