# Supplementary material for: Biomonitoring of 19 Mycotoxins in Plasma from Food-Producing Animals (Cattle, Poultry, Pigs, and Sheep)
Source: Toxins (Basel). 2023 Apr 18;15(4):295. doi: 10.3390/toxins15040295 (PMC10144229; doi:10.3390/toxins15040295)
Supplement: Supplementary file 1 [file toxins-15-00295-s001.zip › toxins-2319902-supplementary.pdf]

toxins

MDPI

Table S1. Linearity results for the matrix-matched calibration curves obtained for each mycotoxin in plasma with enzymatic treatment.

Mycotoxin	Cattle			Pigs			Poultry			Sheep		
	R^2	Equation	RE * (%)	R^2	Equation	RE (%)	R^2	Equation	RE (%)	R^2	Equation	RE (%)
DOM-1	0.9975	y = 13.54x − 7.62	13.4	0.9938	y = 14.48x − 0.16	18.0	0.9969	y = 13.76x − 35.26	15.6	0.9981	y = 13.24x + 5.29	11.0
AFG2	0.9976	y = 129.62x − 7.70	20.0	0.9982	y = 134.18x − 53.68	18.0	0.9968	y = 138.17x − 20.76	12.7	0.9982	y = 136.67x − 5.68	9.3
AFM1	0.9996	y = 110.83x − 51.82	18.3	0.9990	y = 128.36x + 14.24	10.4	0.9921	y = 129.36x − 4.56	14.2	0.9987	y = 131.53x + 9.23	18.1
AFG1	0.9982	y = 302.26x − 44.49	17.4	0.9991	y = 362.45x − 24.14	12.1	0.9959	y = 383.54x − 18.36	12.7	0.9981	y = 370.44x − 30.74	9.8
AFB2	0.9983	y = 370.00x − 7.57	14.4	0.9908	y = 351.68x + 7.68	11.5	0.9956	y = 355.95x − 5.48	13.0	0.9982	y = 350.83x − 56.01	18.3
AFB1	0.9910	y = 535.29x − 30.79	14.9	0.9982	y = 541.75x − 42.78	11.2	0.9979	y = 515.14x + 0.18	10.0	0.9927	y = 518.57x + 13.22	18.8
HT-2	0.9995	y = 18.19x − 19.69	6.3	0.9992	y = 15.76x + 29.27	10.0	0.9986	y = 17.36x + 19.28	11.1	0.9981	y = 16.83x − 19.45	13.2
OTB	0.9993	y = 95.75x + 15.87	13.6	0.9975	y = 116.42x − 17.19	11.7	0.9988	y = 102.94x − 1.02	11.0	0.9971	y = 99.93x + 13.89	7.8
T-2	0.9982	y = 97.85x − 5.70	6.2	0.9990	y = 99.51x + 32.98	11.2	0.9952	y = 105.76x + 0.04	11.4	0.9977	y = 102.41x + 20.47	13.8
ZEA	0.9993	y = 24.27x − 38.88	14.9	0.9977	y = 26.92x − 47.71	18.4	0.9933	y = 25.11x − 20.13	14.7	0.9983	y = 27.01x − 10.91	5.3
OTA-d_5	0.9958	y = 66.04x − 11.81	9.8	0.9994	y = 50.94x − 11.47	9.9	0.9994	y = 62.06x − 56.12	16.6	0.9931	y = 55.81x − 54.46	11.3
STER	0.9900	y = 123.45x + 2.38	14.7	0.9945	y = 97.23x + 27.59	15.3	0.9906	y = 100.32x − 16.49	15.7	0.9983	y = 109.78x − 31.39	15.2
NIV	0.9976	y = 3.56x − 37.95	19.0	0.9979	y = 3.44x − 34.18	15.4	0.9937	y = 3.23x + 23.64	13.9	0.9993	y = 3.64x − 45.56	17.9
DON	0.9910	y = 7.66x-6.97	11.3	0.9994	y = 7.32x + 10.54	13.3	0.9993	y = 7.65x − 12.23	12.7	0.9979	y = 8.84x − 51.71	19.0
FUS-X	0.9937	y = 11.12x − 12.79	10.4	0.9959	y = 10.43x − 41.29	12.3	0.9974	y = 10.01x − 4.61	14.2	0.9994	y = 10.05x − 18.90	4.2
NEO	0.9908	y = 88.03x − 0.41	15.0	0.9967	y = 77.09x + 30.83	14.0	0.9983	y = 87.48x − 9.06	13.8	0.9980	y = 91.31x − 22.35	11.8
3-ADON	0.9992	y = 39.40x − 25.20	11.5	0.9980	y = 39.57x − 18.48	12.1	0.9975	y = 39.99x + 19.09	14.6	0.9954	y = 40.91x − 10.73	13.0
15-ADON	0.9963	y = 25.89x − 11.45	14.1	0.9993	y = 24.77x − 22.41	10.4	0.9954	y = 23.19x + 23.94	13.2	0.9907	y = 27.08x − 10.15	13.6
DAS	0.9904	y = 100.45x − 16.12	10.5	0.9985	y = 97.36x + 0.75	12.2	0.9994	y = 100.61x − 27.23	8.5	0.9991	y = 103.69x + 8.21	11.1

* RE(%): The maximum value of relative errors of back calculated concentrations is shown.

toxins

MDPI

Table S2: Qualification/quantification peak areas ratio and retention times (min) for each one of the mycotoxins in a standard (fortified mobile phase) and a calibrator (fortified pig plasma) at LOQ level without enzymatic treatment.

Mycotoxin	q/Q Standard	q/Q Calibrator	RE (%)	RT Standard (min)	RT Calibrator (min)	RE (%)
DOM-1	84.3	84.5	0.2	1.4	1.5	1.3
AFG2	79.2	80.6	1.7	2.4	2.4	0.6
AFM1	82.7	80.8	2.3	2.5	2.5	0.9
AFG1	76.1	83.9	9.3	2.9	2.9	0.8
AFB2	101.3	105.1	3.6	3.7	3.6	1.5
AFB1	68.5	73.6	6.9	4.5	4.5	0.2
HT-2	61.1	66.5	8.1	9.6	9.6	0.1
OTB	44.5	46.1	3.5	11.6	11.5	0.2
T-2	71.6	70.2	2.0	13.2	13.2	0.2
ZEA	80.2	84.8	5.4	14.8	14.8	0.1
OTA-d_5	64.8	65.5	1.1	15.7	15.7	0.2
STER	79.7	85.9	7.2	15.8	15.8	0.0
NIV	95.1	95.6	0.5	4.1	4.1	0.2
DON	86.7	90.3	4.1	2.4	2.4	0.6
FUS-X	77,9	74.7	4.1	8.0	8.0	0.1
NEO	81.2	85.3	4.8	8.7	8.7	0.0
3-ADON	81.4	76.3	6.3	10.0	9.9	0.5
15-ADON	91.8	90.9	1.0	10.1	10.1	0.3
DAS	74,55	67.3	9.8	14.0	14.0	0.2

q: Transition of qualification. Q: Transition of quantification. RE: relative error. RT: retention time.

Table S3. Matrix effect and recovery (at LOQ), and precision, and accuracy in fortified pig plasma and with enzymatic treatment.

Mycotoxin	Matrix effect (%)	Recovery (%)	Precision (RSD%) (n=3)			Accuracy (RE%) n=3		
			LOQ *	6× LOQ	30× LOQ	LOQ	6× LOQ	30× LOQ
DOM-1	98.4	85.5	9.1	4.5	1.5	2.0	4.6	11.8
AFG2	85.8	95.2	13.5	4.7	3.2	6.5	5.3	2.6
AFM1	96.3	91.4	11.6	2.9	1.9	5.2	4.2	3.1
AFG1	88.4	95.0	10.5	2.4	2.3	3.5	7.7	4.0
AFB2	88.5	91.7	9.4	2.7	4.3	6.4	5.1	1.9
AFB1	89.1	95.2	9.9	2.9	4.0	5.6	5.1	3.1
HT-2	102.5	90.2	8.7	2.6	2.3	10.8	4.5	0.8
OTB	87.6	85.2	9.4	4.4	2.2	3.3	3.3	1.1
T-2	85.3	95.4	8.7	1.5	1.8	9.7	4.8	5.9
ZEA	93.7	100.0	7.2	2.1	1.6	6.3	4.7	4.7
OTA-d_5	84.7	91.5	6.2	1.6	2.5	5.4	6.7	1.8
STER	85.2	81.5	8.6	2.1	5.4	7.1	7.6	3.9
NIV	93.9	95.2	6.8	3.7	4.5	12.6	8.6	4.1
DON	85.0	84.9	12.6	2.7	5.9	8.0	3.0	2.2
FUS-X	109.7	112.8	13.2	7.0	5.3	11.0	5.2	9.1
NEO	90.1	85.7	7.5	4.2	3.5	9.8	2.6	2.5
3-ADON	113.9	87.8	12.9	5.3	3.4	2.9	1.2	1.4
15-ADON	102.1	112.8	7.6	5.1	4.0	3.8	7.0	2.6
DAS	87.2	87.8	12.1	5.2	3.5	6.2	3.5	3.7

* LOQ: limit of quantification. RSD: relative standard deviation, RE: relative error.

Table S4. Results obtained for STER (ng/mL) in samples with enzymatic treatment of the plasma.

Sample	Cattle	Pigs	Poultry	Sheep
1	2.57	*0.91*	*0.83*	*0.89*
2	3.18	1.47	2.78	2.93
3	1.59	2.28	1.16	3.60
4	1.30	1.33	2.91	1.08
5	3.70	2.40	*0.77*	*0.97*
6	2.15	1.62	1.32	1.38
7	1.02	1.99	1.80	*0.96*
8	1.77	2.74	1.30	3.04
9	1.61	3.93	3.55	1.44
10	1.19	2.91	1.15	*0.82*
11	*0.97*	4.65	3.09	4.91
12	1.50	4.71	2.07	3.45
13	1.13	4.46	1.59	1.51
14	1.37	2.15	2.10	3.54
15	3.35	4.41	2.55	3.80
16	3.90	1.12	1.06	*0.81*
17	4.25	4.96	3.91	4.66
18	3.41	3.20	3.82	1.67
19	4.07	4.71	*0.70*	3.72
20	3.74	1.92	*0.81*	3.53

LOD: 0.2 ng/mL, LOQ: 1.0 ng/mL. Italics >LOD and <LOQ value.

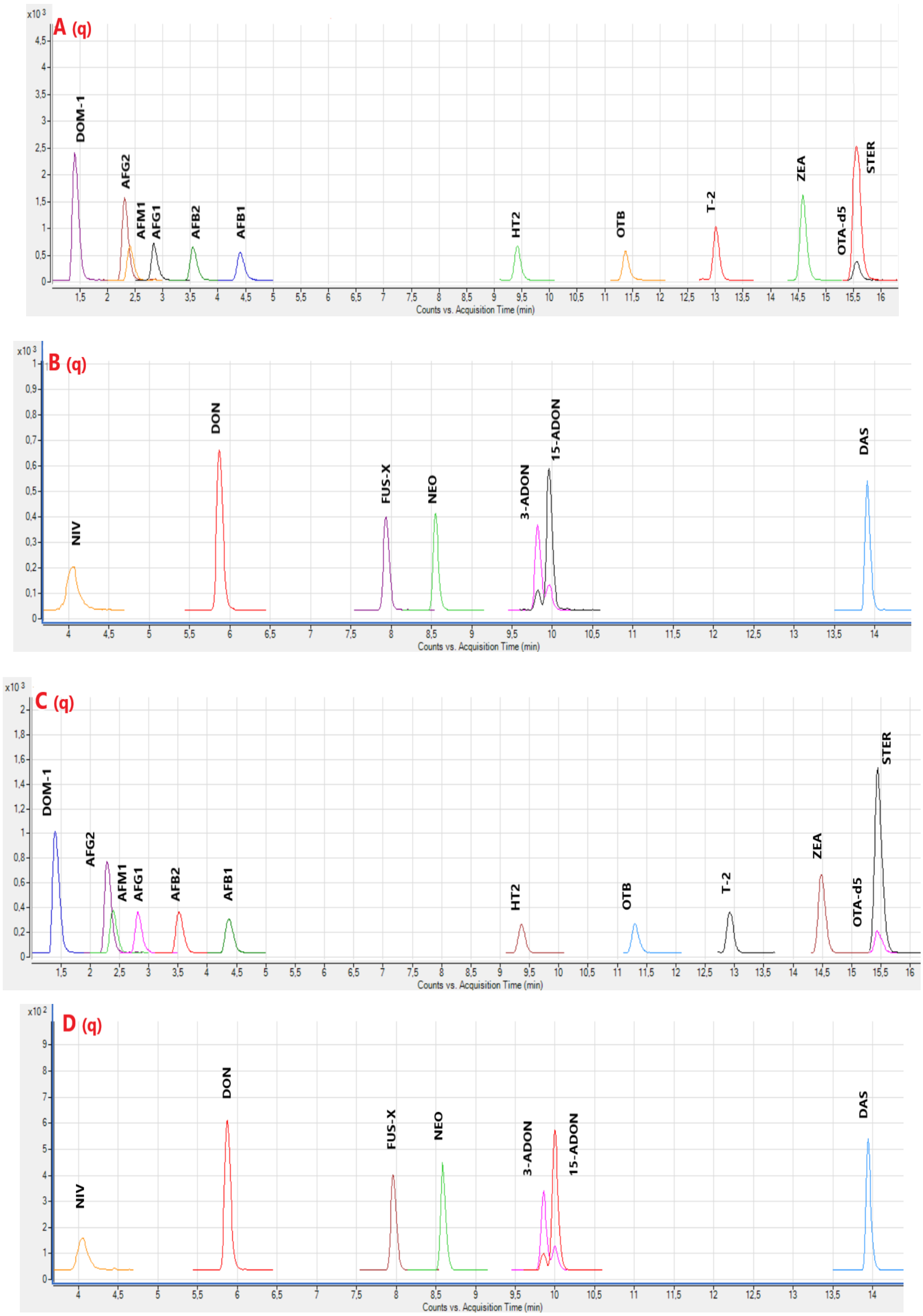
A (q)
x10 3
DOM-1
AFG2
AFM1
AFG1
AFB2
AFB1
HT2
OTB
T-2
ZEA
OTA-d5
STER
Counts vs. Acquisition Time (min)
B (q)
x10 3
NIV
DON
FUS-X
NEO
3-ADON
15-ADON
DAS
Counts vs. Acquisition Time (min)
C (q)
x10 3
DOM-1
AFG2
AFM1
AFG1
AFB2
AFB1
HT2
OTB
T-2
ZEA
OTA-d5
STER
Counts vs. Acquisition Time (min)
D (q)
x10 2
NIV
DON
FUS-X
NEO
3-ADON
15-ADON
DAS
Counts vs. Acquisition Time (min)

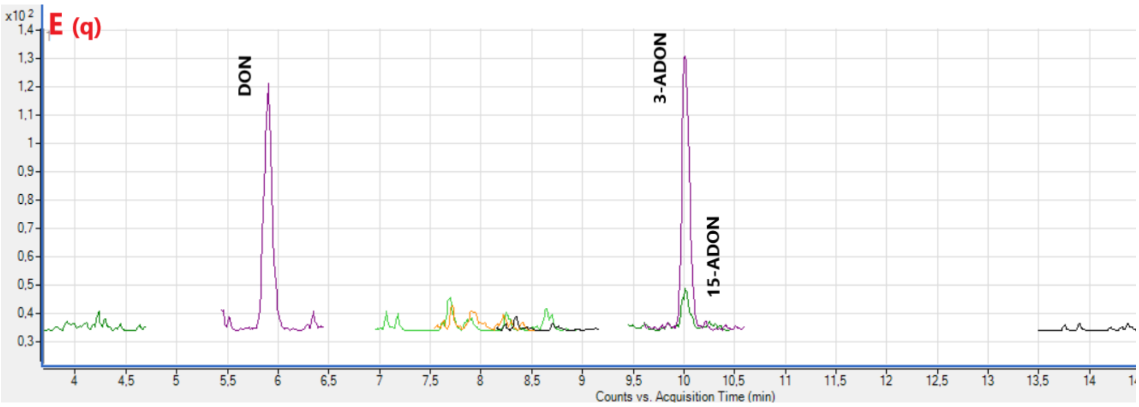

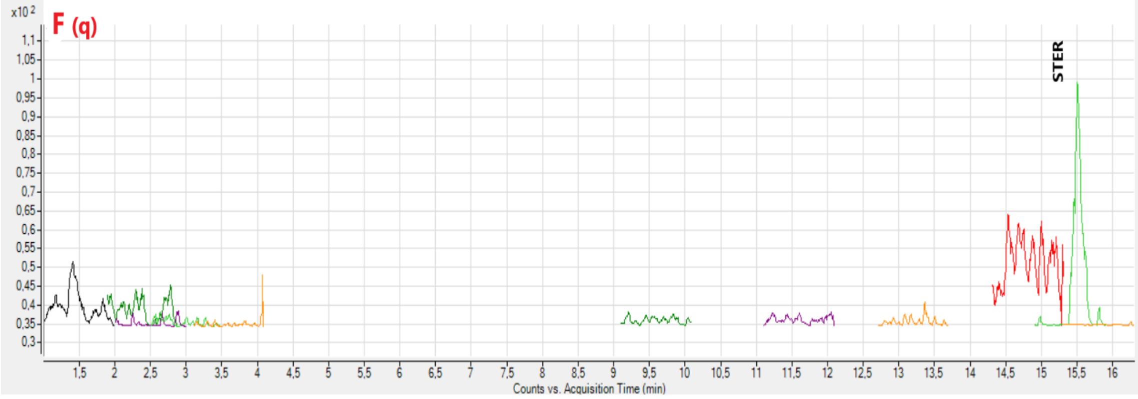

Figure S1. (**A**) Superposed **c**hromatograms (q) obtained from a calibrator at 10× LOQ level without enzymatic treatment (mycotoxins from group I). (**B**) Superposed chromatograms (q) obtained from a calibrator at 10× LOQ levels without enzymatic treatment (mycotoxins from group II). (**C**) Superposed chromatograms (q) obtained from a calibrator at 10× LOQ levels with enzymatic treatment (mycotoxins from group I). (**D**) Superposed chromatograms (q) obtained from a calibrator at 10× LOQ levels with enzymatic treatment (mycotoxins from group II). (**E**) Superposed chromatograms (q) obtained from a sample of poultry plasma without enzymatic treatment containing DON, 3- and 15- ADON). (**F**) Superposed chromatograms (q) from a sample of poultry plasma with enzymatic treatment containing STER.
